# Silica Spheres Functionalized with Silver and Bismuth Nanoparticles—Antibacterial Activity Against Clinically Relevant Bacterial Pathogens

**DOI:** 10.3390/ijms262010203

**Published:** 2025-10-20

**Authors:** Marcin Gajek, Karolina Klesiewicz, Maria Biegun-Żurowska, Paula Parreira, Magdalena Ziąbka, Agnieszka Różycka, Alicja Rapacz-Kmita

**Affiliations:** 1 Faculty of Materials Science and Ceramics, AGH University of Krakow, Av. Mickiewicza 30, 30-059 Krakow, Poland; biegun@agh.edu.pl (M.B.-Ż.); ziabka@agh.edu.pl (M.Z.); ar@agh.edu.pl (A.R.); kmita@agh.edu.pl (A.R.-K.); 2 Department of Pharmaceutical Microbiology, Faculty of Pharmacy, Jagiellonian University Medical College, Medyczna 9, 30-688 Krakow, Poland; 3 i3S—Instituto de Investigação e Inovação em Saúde, Rua Alfredo Allen, 208, 4200-135 Porto, Portugal; parreira@i3s.up.pt

**Keywords:** silver nanoparticles, bismuth nanoparticles, silica spheres, antimicrobial activity

## Abstract

The aim of the study was to develop hybrid nanomaterials based on monodisperse silica spheres as carriers for silver nanoparticles (AgNPs) or bismuth nanoparticles (BiNPs) and to evaluate their antimicrobial properties. Silica spheres were synthesized using a modified Stöber method, either unmodified or functionalized with (3-aminopropyl)triethoxysilane (APTES), prior to AgNP or BiNP deposition. The materials were characterized by scanning electron microscopy (SEM), transmission electron microscopy (TEM), X-ray diffraction (XRD), inductively coupled plasma optical emission spectroscopy (ICP-OES), and zeta potential measurements, while antimicrobial activity was assessed by microdilution against Gram-positive (*Staphylococcus aureus*, *Staphylococcus epidermidis*, *Enterococcus faecalis*, *Enterococcus faecium*) and Gram-negative bacteria (*Escherichia coli*, *Pseudomonas aeruginosa*), with *Helicobacter pylori* as a clinical model. The results show that both SiO_2_-AgNP and SiO_2_-BiNP composites completely inhibited *H. pylori* and showed high activity against other pathogens, although *P. aeruginosa* remained less susceptible. Functionalization of AgNP-coated samples with APTES promoted uniform distribution of AgNPs, with the minimum bactericidal concentration (MBC) to minimum inhibitory concentration (MIC) ratios ranging from 1 to 4, confirming a bactericidal rather than bacteriostatic effect. In contrast, BiNP-coated samples without APTES exhibited lower MIC values from 74 to 595 μg mL^−1^, consistent with increased Bi^3+^ release from amorphous phases. This indicates the antimicrobial potential, highlighting the role of surface functionalization in regulating ion release and biological performance, and suggesting applications in the biomedical and food industries.

## 1. Introduction

Advances in medicine and pharmacology are improving quality of life, but the problem of bacterial resistance to antibiotics continues to grow. Microorganisms, which have always accompanied humans, are developing defense mechanisms against drugs, making infections that were once easy to control increasingly difficult to treat. As a result, chronic and recurrent diseases emerge that do not respond to standard therapies [1], and according to the World Health Organization (WHO), antibiotic resistance and infections caused by multidrug-resistant organisms (MDRO) are one of the ten greatest threats to public health [2]. It is estimated that by 2050, the number of deaths due to infections with multidrug-resistant pathogens may reach 10 million annually, exceeding the number of deaths due to cancer [3]. The increasing resistance of microorganisms, the limited therapeutic options, and the difficulties in developing a new class of drugs with a mechanism of action different from those currently known prompt the search for alternative strategies to counteract resistance.

One such promising alternative strategy that can provide stand-alone solutions or be a complement to traditionally used antibiotics is the use of nanometric materials exhibiting antimicrobial properties, especially in the fight against multidrug-resistant (MDR) strains. Among such nanomaterials, silver and bismuth in the form of silver nanoparticles (AgNPs) and bismuth nanoparticles (BiNPs) are of particular interest due to their broad-spectrum antimicrobial activity and relatively low risk of inducing resistance. These metals interact with bacteria via physicochemical pathways, independently of classical antibiotic mechanisms. For example, silver damages bacterial cell membranes, disrupts enzyme function, destroys genetic material and generates reactive oxygen species [4,5], making it effective against both Gram-positive and Gram-negative bacteria [6,7]. Bismuth and its compounds, although used less frequently, demonstrate activity against various resistant strains of bacteria, including *Escherichia coli* and *Staphylococcus aureus* [8], but they are also used, among others, in the standard treatment of stomach infections caused by *Helicobacter pylori* [9]. The mechanism of bismuth’s action in this context is explained by its interference with enzymatic protein function, which causes destabilization of the bacterial cell membrane, and its influence on the cellular redox system, and although it is relatively well understood, it is still the subject of ongoing research [8]. Studies have also shown that bismuth (III) complexes with 8-hydroxyquinoline are effective against both Gram-positive and Gram-negative bacteria, with minimal toxicity to human cells [10]. In turn, bismuth nanoparticles (BiNPs), characterized by chemical stability, moderate toxicity, and high specific surface area [8,11,12], are not only being investigated in the context of cancer therapy, medical imaging, and as drug carriers [13], but also demonstrate activity against multidrug-resistant strains, interacting directly with the bacterial cell wall, limiting biofilm formation, generating reactive oxygen species, and influencing the host’s immune response [14,15].

Nanoparticles (NPs) differ in their properties from microparticles, primarily due to their larger specific surface area. As a result, they exhibit greater reactivity and effectiveness, especially in biological applications; therefore silver and bismuth in the form of nanoparticles may exhibit many times more potent antimicrobial activity than their larger counterparts [5,16]. An increasing number of studies explores the use of nanomaterials in combating bacterial infections, with silver nanoparticles already being used commercially in wound dressings, medical coatings, and disinfectants [17], while bismuth has recently emerged as a component of materials with low toxicity and high-efficacy materials [18].

A problem with nanomaterial applications, however, is their aggregation, which reduces the effective surface area. This can limit the excessive activity that can sometimes lead to toxicity to host cells, especially with long-term exposure [17,19], but in general, this results in a significant reduction in antibacterial activity. One solution to limit aggregation and enable controlled release of metal ions is the stabilization of nanoparticles using carriers such as polymers, proteins, chitosan, alginate, and amorphous silica [13,16,20]. Combining metal with a suitable carrier, such as silica nanospheres, is a novel approach that integrates knowledge from chemistry, materials science, and microbiology, enabling the development of durable, active, and safe antimicrobial materials in response to the growing threat of bacterial resistance to available therapies. The Stöber method [20,21,22,23] has proven particularly useful for obtaining spherical silica, enabling the synthesis of silica (SiO_2_) nanospheres with controlled size [24]. The resulting particles are non-toxic, chemically stable, disperse well in solutions, forming homogeneous suspensions, and can be easily modified. As a result, they provide a good scaffold for the deposition of metals such as silver and bismuth, while silica prevents metal aggregation and can influence the way they interact with microorganisms, e.g., by facilitating contact with the bacterial cell membrane or regulating the rate of ion release from the metal [25].

This study aimed to develop a stable form of nanomaterial combining a silica carrier with an active metal, as a potential strategy for limiting the transmission of clinical pathogens. For this purpose, SiO_2_ nanoparticles with silver or bismuth nanoparticles deposited on their surface were obtained. The conducted studies, including the evaluation of material morphology (scanning electron microscopy (SEM), transmission electron microscopy (TEM)), phase composition (X-ray diffraction (XRD)), elemental composition (inductively coupled plasma optical emission spectroscopy (ICP-OES)), and zeta potential analysis, as well as antimicrobial tests against Gram-positive and Gram-negative bacterial strains with determination of the key minimum bactericidal concentration (MBC) to minimum inhibitory concentration (MIC) ratio, allowed for the assessment of their properties in terms of designing materials with effective antibacterial action. Furthermore, the nanoparticles showing the most promising activity against *H. pylori* were tested for their cytocompatibility with two human gastric cancer cell lines, MKN28 (gastric carcinoma) and AGS (gastric adenocarcinoma).

## 2. Results and Discussion

### 2.1. Morphological Characterization of Silica Spheres Coated with Silver and Bismuth Nanoparticles

**Figure 1:**
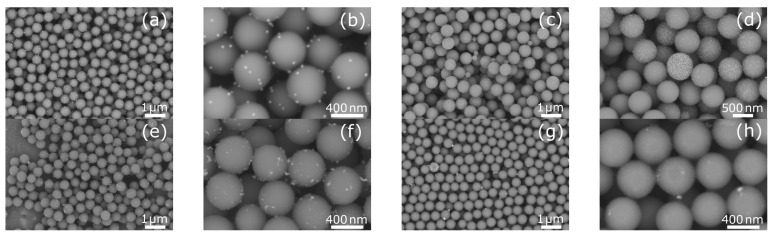
Scanning electron microscopy (SEM) images of silica spheres coated with silver or bismuth nanoparticles: (**a**,**b**) SiO_2_-AgNPs1 (Ag, no (3-aminopropyl)triethoxysilane (APTES), 2.04 wt.% Ag); (**c**,**d**) SiO_2_-AgNPs2 (Ag, with APTES, 2.57 wt.% Ag); (**e**,**f**) SiO_2_-BiNPs1 (Bi, no APTES, 2.38 wt.% Bi); (**g**,**h**) SiO_2_-BiNPs2 (Bi, with APTES, 4.52 wt.% Bi).

**Figure 2:**
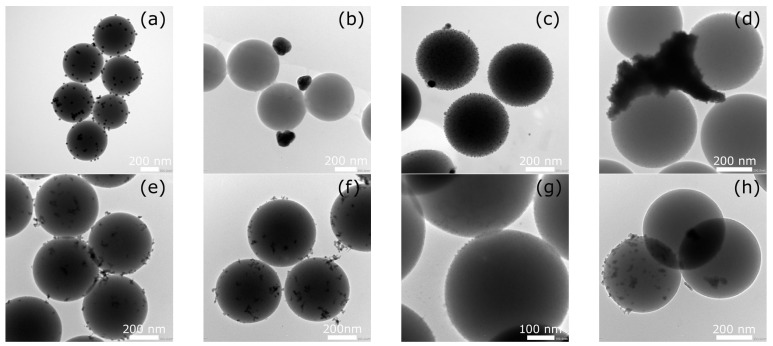
Transmission electron microscopy (TEM) images of silica spheres coated with silver or bismuth nanoparticles: (**a**,**b**) SiO_2_-AgNPs1 (Ag, no APTES, 2.04 wt.% Ag); (**c**,**d**) SiO_2_-AgNPs2 (Ag, with APTES, 2.57 wt.% Ag); (**e**,**f**) SiO_2_-BiNPs1 (Bi, no APTES, 2.38 wt.% Bi); (**g**,**h**) SiO_2_-BiNPs2 (Bi, with APTES, 4.52 wt.% Bi).

Silver or bismuth nanoparticles were deposited in the designed materials on silica spheres constituting a stable substrate with an average size of about 500 nm, confirmed by SEM and TEM image analysis. SEM observations (Figure 1) showed that AgNPs deposited on the surface of SiO_2_ both without and with prior surface functionalization using (3-aminopropyl)triethoxysilane (APTES) were uniformly distributed. However, in the SiO_2_-AgNPs1 and SiO_2_-AgNPs2 samples, agglomerates of metal nanoparticles were also visible in some areas, which was also confirmed by TEM images (Figure 2). It should be emphasized that the TEM images presented in Figure 2 for each sample show both representative areas in which nanoparticles were evenly distributed (Figure 2a,c,g) as well as those in which local agglomeration was observed (Figure 2b,d,h). In the case of the SiO_2_-BiNPs1 sample (Figure 2e,f), no obvious agglomerates were observed, whereas for the SiO_2_-BiNPs2 sample the representative area (Figure 2g) shows a uniform distribution, and a rare region with slight local clusters is shown in Figure 2h. The purposeful selection of such images was aimed at showing both the dominant pattern of nanoparticle distribution and the less frequent, but important from the point of view of material characterization, cases of their local aggregation. On the surface of the SiO_2_-AgNPs1 materials, crystallites of approximately 20 nm in size were visible, statistically dispersed over the silica surface. Additional surface modification of SiO_2_ using APTES (SiO_2_-AgNPs2) favored the formation of very small, well-defined AgNPs with sizes of approximately 5–10 nm (Figure 1 and Figure 2). The presence of larger clusters and agglomerates of silver on the SiO_2_ surface was influenced by the material preparation process, in particular the washing/purification steps and the redispersion of the particles in deionized water using ultrasonication. Functionalization with APTES mainly influenced the uniform distribution of nanoparticles and their size distribution, which led to the formation of more numerous, smaller Ag nanoparticles while maintaining their strong bond to the silica support.

In the case of bismuth-containing samples, a slightly different distribution pattern of BiNPs on the surface of SiO_2_ spheres was observed. For the SiO_2_-BiNPs1 material (without APTES, 2.38 wt.% Bi), in which polyvinylpyrrolidone (PVP) was added directly to the SiO_2_ suspension before the bismuth solution was introduced, both SEM and TEM analysis revealed individual crystallites as well as numerous bismuth aggregates deposited on the surface of the SiO_2_ spheres. These aggregates consist of several connected primary particles with estimated sizes below 5 nm, forming structures with total dimensions of 5–25 nm. Their amorphous, irregular morphology is associated with local surface development, which may facilitate faster release of Bi^3+^ ions. Importantly, these aggregates did not undergo significant disintegration under the applied sonication, which may indicate the durable nature of their internal connections.

For the SiO_2_-BiNPs2 material (APTES-modified, 4.52 wt.% Bi), SEM and TEM images (Figure 1 and Figure 2) showed a continuous layer of bismuth nanoparticles with a size of ≤5 nm, uniformly distributed over the surface of the SiO_2_ spheres. Such a uniform distribution can be attributed to the effect of SiO_2_ surface functionalization with amino groups derived from APTES. Additionally, this effect may have been enhanced by the presence of PVP, which, during the deposition stage, was first added to the bismuth solution and then introduced into the SiO_2_ sphere suspension.

### 2.2. Phase Composition

**Figure 3:**
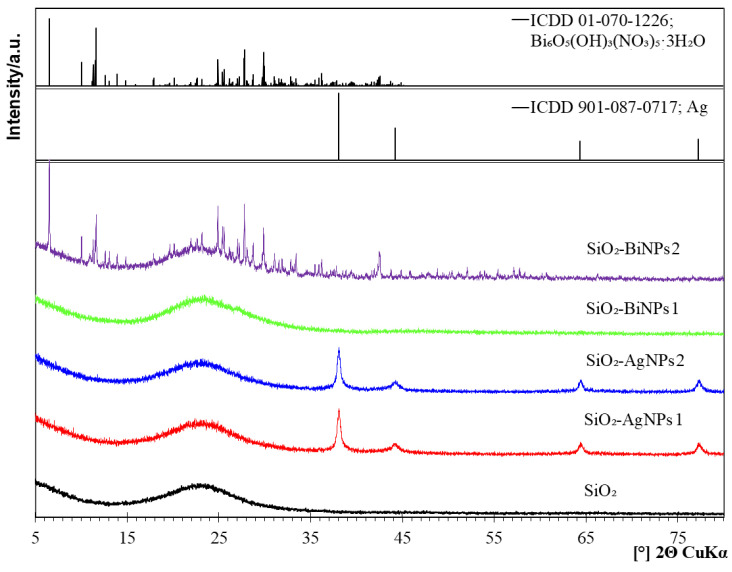
X-ray diffraction patterns of pure silica (SiO_2_) spheres and silica spheres functionalized with Ag and Bi nanoparticles. Reference profiles from the International Centre for Diffraction Data (ICDD) are overlaid: metallic silver (01-087-0717) and Bi_6_O_5_(OH)_3_(NO_3_)_5_·3(H_2_O) (01-070-1226).

X-ray diffractograms presented in Figure 3 confirm the presence of metallic silver (Ag) in the SiO_2_-AgNPs1 and SiO_2_-AgNPs2 materials, and the distinct diffraction peaks observed at 2θ values of 38.1°, 44.3°, 64.4°, and 77.4° correspond to the (111), (200), (220), and (311) crystallographic planes of face-centered cubic (FCC) silver, belonging to the Fm-3m space group (International Centre for Diffraction Data (ICDD) PDF 01-087-0717). The X-ray diffraction (XRD) pattern of purified SiO_2_ spheres, recorded after synthesis, shows a broad, diffuse peak in the 2θ angle range of 15–35°, confirming their amorphous structure. It also provides a reference point enabling unambiguous identification of diffraction peaks originating from metallic silver and bismuth containing phases in the functionalized samples. The positions and intensities of the reflections indicate a high degree of crystallinity of silver and confirm its presence on the surface of the SiO_2_ spheres. At the same time, a broad elevated background observed in the 2θ range of 15–35° is characteristic of amorphous silica, which is consistent with the expected structure of the support matrix.

In the SiO_2_-BiNPs1 material, despite the confirmed presence of BiNPs in SEM and TEM images (Figure 1 and Figure 2), XRD analysis did not reveal diffraction peaks originating from crystalline bismuth phases. The absence of distinct signals may result from the amorphous nature of the BiNPs, their very low degree of crystallinity, or the presence of exceptionally small and dispersed nanoparticles (with sizes below the detection limit, i.e., <3–5 nm). Additionally, any weak crystalline signal may have been masked by the amorphous silica background, which dominates in the analyzed material.

In the SiO_2_-BiNPs2 sample, reflections assigned to bismuth oxide nitrate hydroxide hydrate Bi_6_O_5_(OH)_3_(NO_3_)_5_·3(H_2_O) were identified, in accordance with ICDD card 01-070-1226. This phase crystallizes in the monoclinic system and belongs to the class of layered compounds containing Bi^3+^ ions coordinated with oxygen atoms, hydroxyl groups, and nitrate ions [26,27]. The formation may be explained by incomplete reduction of Bi^3+^ ions due to limited access of the sodium borohydride (NaBH_4_) reducing agent to all metal ions, especially in the presence of PVP and amino groups from APTES. Rapid nucleation of bismuth on the surface of SiO_2_ may have led to local entrapment of regions with excess Bi^3+^ and nitrate ions, promoting partial hydrolysis and stabilization of the Bi_6_O_5_(OH)_3_(NO_3_)_5_·3(H_2_O) phase. This phase may constitute only part of the total amount of bismuth deposited on the SiO_2_ surface and may act as a reservoir of Bi^3+^ ions gradually released during contact with bacterial cells. Part of the bismuth remains in a dispersed, relatively amorphous form or as very small crystallites, which may also contribute to its gradual release, thereby inducing a strong antibacterial effect [28,29].

ICP-OES analysis confirmed the presence of metal in all samples, even in the case of SiO_2_-BiNPs1, where no diffraction peaks were observed. The bismuth content was 2.38 wt.% relative to SiO_2_ for the SiO_2_-BiNPs1 sample, and 4.52 wt.% for SiO_2_-BiNPs2. For the silver-containing samples, 2.04 wt.% Ag was obtained for SiO_2_-AgNPs1 and 2.57 wt.% Ag for SiO_2_-AgNPs2. These data, summarized in Table 1, confirm the effectiveness of metal deposition regardless of their structural form and indicate slightly higher efficiency in the presence of amino groups derived from APTES.

### 2.3. Zeta Potential and Suspension Stability

All samples exhibited a negative zeta potential, which is typical for silica with silanol groups, and the highest absolute values of zeta potential (−38.4 mV for SiO_2_-AgNPs1, −36.1 mV for SiO_2_-AgNPs2, and −33.6 mV for SiO_2_-BiNPs1) indicate good colloidal stability and limited tendency toward aggregation. The distinctly lower zeta potential observed for the SiO_2_-BiNPs2 sample (−5.9 mV) may result from the presence of amino groups introduced during APTES functionalization and the formation of a partially reduced oxide-nitrate layer. The sedimentation of particles observed over time was reversible and likely reflected particle density and size rather than true destabilization of the dispersion, and the suspensions readily regained homogeneity after gentle mixing.

### 2.4. Antibacterial Efficacy of Nanoparticles

The antibacterial activity of the nanocomposites (silica spheres decorated with silver and bismuth nanoparticles) was assessed against a panel of clinically relevant Gram-positive and Gram-negative bacterial strains. The tested pathogens included *S. aureus* (American Type Culture Collection (ATCC^®^) 25923, ATCC^®^ methicillin-resistant *Staphylococcus aureus* (MRSA)), *S. epidermidis* (ATCC^®^ 28212), *E. faecalis* (ATCC^®^ 29212), *E. faecium* vancomycin-resistant (VRE) (ATCC^®^ 700221), *E. coli* (ATCC^®^ 25922), *P. aeruginosa* (ATCC^®^ 27853) and *H. pylori* J99 (ATCC^®^ 700824^TM^).

Both variants of silica spheres with deposited AgNPs (SiO_2_-AgNPs1) (Ag, 1020 µg mL^−1^) and SiO_2_-AgNPs2 (Ag, 1285 µg mL^−1^) showed moderate antibacterial activity. SiO_2_-AgNPs1 revealed MIC values ranging from 127.5 µg mL^−1^ against *S. epidermidis* up to 510 µg mL^−1^ for the least susceptible Gram-positive strains (*E. faecalis*). The activity against Gram-negative bacteria was significantly lower, with MIC equal to or higher than 510 µg mL^−1^. A similar trend was observed for SiO_2_-AgNPs2. The lowest MIC values were revealed against Gram-positive bacteria, with *S. epidermidis* being the most susceptible strain (MIC = 160.6 µg mL^−1^), while for the remaining Gram-positive strains the MIC reached 321.2 µg mL^−1^. In contrast, the activity against Gram-negative bacteria was markedly reduced, with an MIC of 642.5 µg mL^−1^ for *E. coli* and >642.5 µg mL^−1^ for *P. aeruginosa*. The analysis of the bactericidal versus bacteriostatic potential of silver-based nanomaterials, based on the obtained MBC values for both tested SiO_2_-AgNPs, revealed MBC/MIC ratios ranging from 1 to 4, thus confirming their bactericidal activity against Gram-positive bacteria. For Gram-negative bacteria, however, determination of the MBC/MIC ratio was not possible, as the MBC values exceeded the highest concentrations tested (Table 2).

In parallel, silica spheres coated with bismuth nanoparticles (SiO_2_-BiNPs) were examined to assess their antibacterial potential: SiO_2_-BiNPs1; Bi 1190 μg mL^−1^ and SiO_2_-BiNPs2; Bi 2260 μg mL^−1^. For Gram-positive bacteria, SiO_2_-BiNPs1 exhibited MIC values of 297.5 µg mL^−1^ against *S. aureus* and vancomycin-resistant *Enterococcus*, whereas *S. epidermidis* was markedly more susceptible, with an MIC of 74.4 µg mL^−1^. For Gram-negative bacteria, similarly to silver-based nanocomposites, the bismuth-containing nanocomposites also displayed markedly reduced activity, with MIC values of 595 µg mL^−1^ for *E. coli* and exceeding 595 µg mL^−1^ for *P. aeruginosa.* In comparison, the higher bismuth content in SiO_2_-BiNPs2 improved efficacy for *S. aureus* and *S. epidermidis* with MIC 282.5 µg mL^−1^ and 70.625 µg mL^−1^, respectively. However reduced activity against VRE strain (MIC amounted to 565 µg mL^−1^) and Gram-negative bacteria (for *E. coli* the MIC increased to 1130 µg mL^−1^, and for *P. aeruginosa* exceed this value). The MBC/MIC ratios for both types of SiO_2_-BiNPs indicated a bactericidal effect against Gram-positive bacteria, with values ≤ 4. In contrast, for Gram-negative strains this relationship could not be determined, as the MBC values exceeded the highest concentrations tested.

Overall, *S. epidermidis* proved to be the most susceptible strain to all nanomaterials tested, consistently showing the lowest MIC values across the series. In contrast, VRE and MRSA strains generally required significantly higher concentrations to achieve growth inhibition.

Among Gram-negative pathogens, *E. coli* showed greater susceptibility compared to *P. aeruginosa*, and as expected, the control group of silica spheres (SiO_2_ without metal) showed no activity at the tested concentrations, confirming the key role of the metal component.

Remarkably, among the formulations evaluated, samples with bismuth nanoparticles emerged as the most promising candidates, demonstrating potent activity, particularly against Gram-positive bacteria, including multidrug-resistant strains. These findings align with established multi-target antimicrobial mechanisms reported for AgNPs and BiNPs [5,30,31] and they contextualize the MIC and MBC patterns observed in this study.

In the case of silver-containing samples, the SiO_2_-AgNPs2 sample (with APTES modification) was characterized by lower MIC and MBC values for some strains compared to SiO_2_-AgNPs1. This effect can be explained by the more uniform distribution of AgNPs, smaller particle size (TEM) and tighter anchoring with the silica/APTES interface, which could increase the fraction of bioavailable Ag^+^ in the biological environment and enhance interaction with cells. Although direct ion-release kinetics were not measured, these performance differences are consistent with a higher fraction of available silver in the APTES-functionalized material.

**Figure 4:**
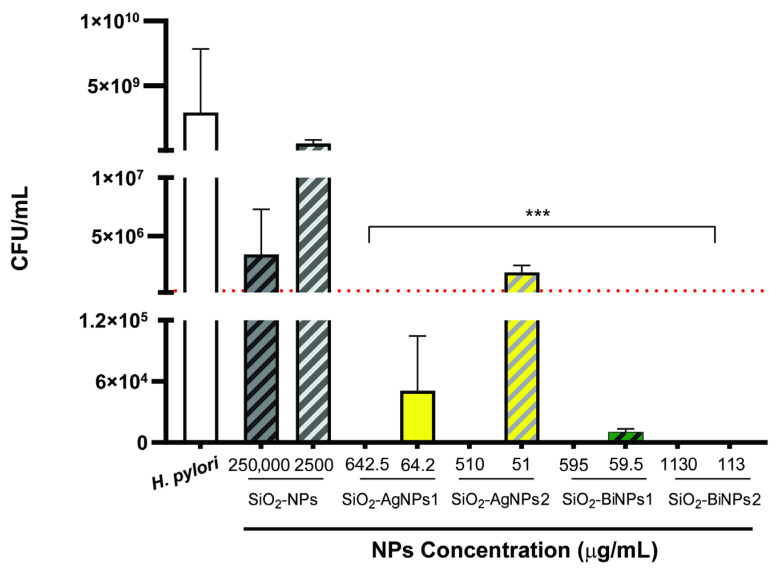
Colony-forming unit (CFU mL^−1^) counts for *H. pylori* J99 after exposure to silver- or bismuth nanoparticles at two different concentrations. Control nanoparticles were silica nanoparticles without functionalization and growth control was *H. pylori* J99 without nanoparticles. Data are represented as mean ± standard deviation. Dotted line represents the bactericidal threshold (3-log10 reduction). ***—statistically significantly different from the growth control (*H. pylori* J99; *p* = 0.0002, One-way analysis of variance (ANOVA) with Kruskal–Wallis test).

For the bismuth-containing samples, SiO_2_-BiNPs1 and SiO_2_-BiNPs2, complete inhibition of *H. pylori* J99 growth was achieved (Figure 4), along with a clear effect against other strains (Table 2). The SiO_2_-BiNPs1 sample, not modified with APTES, exhibited lower MIC values compared to SiO_2_-BiNPs2, which may result from a more amorphous and irregular morphology of the bismuth phase, leading to a faster release of Bi^3+^ ions. The confirmed presence of the Bi_6_O_5_(OH)_3_(NO_3_)_5_·3H_2_O phase in the SiO_2_-BiNPs2 may act as a reservoir of ions while limiting rapid release due to higher structural order.

Interestingly, all tested NPs, except the silica sphere control (SiO_2_ without metal), significantly reduced the growth of *H. pylori* J99 (Figure 4). With the exception of SiO_2_-AgNPs2 at the lowest concentration (51 µg mL^−1^), all treatments were bactericidal against *H. pylori*. Bactericidal activity was defined as a ≥3-log10 reduction in CFU relative to the growth control, that is a 99.9% reduction in bacterial burden. The dotted red line in Figure 4 marks this threshold.

The effect of the NP functionalization with either Ag or Bi to achieve antimicrobial performance is well documented in these results, as approximately 22 times fewer NPs (bare silica nanoparticles at 250,000 µg mL^−1^ versus SiO_2_-BiNPs2 at 1130 µg mL^−1^) are required to achieve antibacterial effect. Overall, bismuth-functionalized nanoparticles appear more promising than silver-functionalized ones, with bismuth’s mechanism of action including binding of Bi^3+^ ions to proteins and disruption of cellular processes, which may explain its high effectiveness against hard-to-treat bacteria such as *H. pylori*. In fact, the last generation therapy against this gastric pathogen is the bismuth quadruple therapy, that consists of a proton pump inhibitor, bismuth, tetracycline and metronidazole, administered for 10–14 days. The addition of bismuth enhances the bactericidal effect, improving eradication rates, with no *H. pylori* resistance to bismuth yet been reported [32,33]. However, relying on antibiotics has severe limitations, and the use of prolonged multi-drug therapeutic schemes increases costs, adverse effects and the risks of drug resistance. At the same time, the gut microbiota balance (dysbiosis) is also altered, resulting in gastrointestinal side effects, such as chronic inflammation and immune dysfunction [33,34]. Here, complete eradication was observed when *H. pylori* was exposed to SiO_2_-BiNPs2, even at the lowest concentration tested (113 µg mL^−1^). Furthermore, oral delivery strategies for gastric settings benefit from the use of bioengineered approaches as NPs, as they allow drugs to overcome gastric bioavailability and effectively reach *H. pylori* infection site (under the gastric mucus layer) [35,36]. Another interesting aspect of further research into nanoparticles (NPs) as new therapeutic routes on the verge of the antibiotic crisis is that most antibiotics resistance mechanisms are not relevant for NPs. This is because their mode of action involves direct contact with the bacterial cell wall, leading to structural changes without the need to penetrate the cell and not influenced by genetic adaptation mechanisms [37]. Although the MIC values herein reported are higher than those usually reported for conventional antibiotics (usually in the 2–64 µg mL^−1^ range), a direct comparison is not possible. Antibiotics are small molecules, with well-defined molecular targets while the nanoparticles antimicrobial effects arise from multiple mechanisms, as reactive oxygen species generation and ion release, and their size largely influences the antimicrobial activity.

**Figure 5:**
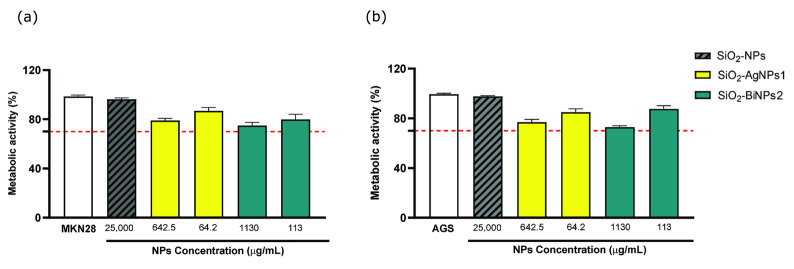
(**a**) MKN28 gastric carcinoma cell line (MKN28) (**b**) AGS gastric adenocarcinoma cell line (AGS) cells metabolic activity after exposure to nanoparticles (NPs) for 24 h. Cells viability tested by resazurin assay. Control nanoparticles are silica NPs at the highest concentration. Negative control: MKN28 and AGS Cells; positive control: cells incubated with 10% (*v*/*v*) solution of 30%V H_2_O_2_. Full Roswell Park Memorial Institute (RPMI) 1640 medium was used as blanks for fluorescence emission subtraction. Data are expressed as mean ± standard deviation. Dotted red line represents the threshold according to ISO 10993-5 standard for cytotoxicity, n = 1 with triplicates.

The cytotoxicity of the most promising NPs against *H. pylori* were then further evaluated by a direct contact assay in accordance with ISO 10993-5 (Figure 5) [38] against two gastric cells lines: the human gastric adenocarcinoma AGS cell line, derived from a human stomach adenocarcinoma and well-known for their strong acid secretory function, and the MKN28 cell line, derived from a human gastric carcinoma and widely used for in vitro infection models [39].

These preliminary results indicate that the tested NPs exhibited a generally non-cytotoxic profile, even at the highest concentration tested, 10× the MBC for *H. pylori*, as cell viability values remained above the 70% threshold in accordance with the ISO standards.

## 3. Materials and Methods

For the synthesis of silica spheres with surface-deposited AgNPs and BiNPs, analytical-grade reagents were used: tetraethoxysilane (TEOS, 99%, Sigma-Aldrich, St. Louis, MO, USA), (3-aminopropyl)triethoxysilane (APTES, 99%, AcroSeal^®^, Thermo Fisher Scientific, Waltham, MA, USA), 25% ammonium hydroxide solution (Avantor Performance Materials Poland, Gliwice, Poland), absolute isopropanol and ethanol (≥99.8%, Avantor Performance Materials Poland), 0.1 M silver nitrate solution (Avantor Performance Materials Poland), sodium borohydride (99%, Thermo Fisher Scientific, Waltham, MA, USA), D-(+)-glucose (≥99%, Chempur, Piekary Śląskie, Poland), polyvinylpyrrolidone K 30 (PVP) (Carl Roth, Karlsruhe, Germany), and bismuth nitrate pentahydrate (≥98%, Avantor Performance Materials Poland). Deionized water with a conductivity of <1 µS·cm^−1^ was used at all stages of synthesis, as well as during the washing of suspensions. Filtration was carried out using polyethersulfone (PES) membranes with a pore size of 0.2 µm (Sarstedt, Nümbrecht, Germany).

### 3.1. Synthesis of Silica Spheres

Spherical SiO_2_ particles were obtained using a modified Stöber method [24] (base-catalyzed sol–gel), with isopropanol as the solvent. A mixture (300 mL) of isopropanol, deionized water, and ammonium hydroxide (molar ratio: isopropanol:NH_4_OH:H_2_O:TEOS—9.43:0.15:9.33:0.45) was stirred using a magnetic stirrer (350 rotation per minute (rpm)) for 15 min at 25 °C, after which TEOS was added in a single portion. The onset of turbidity, indicating the beginning of the synthesis reaction, was observed approximately 7 min after TEOS addition. After 3.5 h of stirring, the resulting spheres were filtered using a PES membrane (0.2 µm) and then washed several times with deionized water to remove reagent residues. After each washing, the sample was redispersed in a fresh portion of deionized water and filtered again. This procedure was repeated multiple times. Finally, the obtained material was dried at 105 °C for 24 h.

### 3.2. Surface Modification of Silica Spheres

A portion of the SiO_2_ spheres obtained in the first stage was subjected to surface functionalization using APTES to introduce amino groups that promote the binding of metal ions. Dried SiO_2_ (1.0 g) was dispersed in a mixture of ethanol and water (97 mL ethanol + 3 mL H_2_O) and ultrasonicated for 3 min (Hielscher Ultrasonics GmbH, Teltow, Germany, 200 W, 24 kHz). The sonication process was carried out with continuous cooling of the vessel (25 °C). After dispersion of the spheres, the suspension was stirred (350 rpm) with the addition of 0.2 mL of APTES for 3 h. Then, the material was filtered, washed with deionized water, and dried at 105 °C for 24 h.

### 3.3. Deposition of Silver Nanoparticles

Samples of silica spheres with silver deposited on the surface were prepared in two variants: without surface modification using APTES (SiO_2_-AgNPs1) and with APTES modification (SiO_2_-AgNPs2). Dried SiO_2_ spheres (0.5 g) were dispersed in 50 mL of deionized water, subjected to ultrasonication for 3 min (Hielscher, 200 W, 24 kHz), and stirred for 30 min using a magnetic stirrer. Then, 3 mL of PVP (10% *m*/*v* solution) was added to the suspension. After 30 min of mixing, a freshly prepared [Ag(NH_3_)_2_]^+^ complex was introduced, and the mixture was stirred for another 60 min. Reduction was carried out with the addition of a glucose solution (2-fold molar excess) for 2 h at 70 °C. The product obtained in this way was filtered, washed with deionized water, and dried at 105 °C.

### 3.4. Deposition of Bismuth Nanoparticles

Silica spheres with surface-deposited bismuth were also prepared in two variants. In the first variant (SiO_2_-BiNPs1, without APTES modification), SiO_2_ spheres (0.5 g) were dispersed in 50 mL of deionized water, sonicated (3 min), and stirred for 30 min. Then, 3 mL of PVP (10% *m*/*v* solution) was added, and the suspension was mixed for another 30 min. In the next step, a freshly prepared 0.01 M solution of Bi(NO_3_)_3_ was introduced and mixed for 60 min. Then, bismuth reduction was carried out using NaBH_4_ (2-fold molar excess) for 2 h at 25 °C. In the second variant (SiO_2_-BiNPs2, with APTES modification), SiO_2_ spheres (0.5 g) were dispersed in 50 mL of deionized water, subjected to sonication (3 min), and stirred for 30 min using a magnetic stirrer. Separately, a fresh solution of Bi(NO_3_)_3_ (0.01 M, pH < 2) was prepared with the addition of 3 mL of PVP (10% *m*/*v* solution), which was introduced into the SiO_2_ suspension and mixed for 60 min. Bismuth reduction was carried out with the addition of NaBH_4_ (2-fold molar excess) for 1 h at 25 °C.

An overview of all obtained nanocomposite variants, including the type of surface functionalization and the intended metal content, is presented in Table 1.

### 3.5. Morphology and Composition

Morphology studies of silica spheres coated with silver and bismuth nanoparticles were performed using a low vacuum, high-resolution scanning electron microscope (SEM) Apreo 2S (Thermo Fisher Scientific, Waltham, MA, USA). Prior to imaging, all samples were coated with a thin conducting layer of carbon (2 nm) and observations were performed under high vacuum conditions using an in-column detector (T1) at an accelerating voltage of 5 kV.

Transmission electron microscopy (TEM) images were obtained using a 200 kV transmission electron microscope JEM-ARM200F NEOARM (JEOL, Tokyo, Japan) equipped with an energy dispersive X-ray spectroscopy (EDS) detector. Samples for SEM and TEM observations were collected directly from the suspension immediately after the synthesis.

Phase composition was determined by X-ray diffraction method (XRD) using an X’Pert Pro diffractometer (Panalytical B.V., Almelo, The Netherlands) with Cu Kα_1_ radiation (λ = 1.5406 Å) and an incident beam monochromator. Patterns were scanned from 5 to 90° 2θ (step size 0.008°, scan time 175 s/step) at 25 °C, and phases were identified using the ICDD PDF database.

Elemental analysis (Ag, Bi) was performed using an iCAP PRO XP Duo spectrometer (Thermo Fisher Scientific, Waltham, MA, USA). Samples (50 mg) were digested in HF/HNO_3_/B_2_O_3_ and diluted to 40 mL with deionized water. Emission lines: Ag 328.068 nm, Bi 223.061 nm. Calibration was performed with multi-element standards in a matched acid matrix.

Zeta potential and mobility were measured with a Zetasizer Nano ZS (Malvern Panalytical Ltd., Malvern, UK), equipped with a dip cell, in deionized water (conductivity < 1 µS·cm^−1^) at natural pH, and the results are reported as the mean of three measurements.

### 3.6. Antibacterial Activity Testing

The antibacterial activity of the silica coating of AgNPs and BiNPs was evaluated against a panel of standard reference strains, including Gram-positive and Gram-negative bacteria. The Gram-positive strains included *Staphylococcus aureus* ATCC^®^ 25923, *Staphylococcus aureus* ATCC^®^ 43300 (methicillin resistant *S. aureus* (MRSA)), *Staphylococcus epidermidis* ATCC^®^ 28212, *Enterococcus faecalis* ATCC^®^ 29212, and *Enterococcus faecium* ATCC^®^ 700221 (vancomycin resistant enterococci (VRE)). The Gram-negative strains included *Escherichia coli* ATCC^®^ 25922 and *Pseudomonas aeruginosa* ATCC^®^ 27853 and *Helicobacter pylori* J99 (ATCC^®^ 700824™).

All bacterial strains, with exception of *H. pylori*, were revived from glycerol stocks by streaking onto Columbia agar with 5% defibrinated sheep blood (Oxoid, Argenta) and incubated aerobically at 37 °C for 18–24 h. *H. pylori* J99 was routinely cultured as described in [39]. Briefly, *H. pylori* were revived from glycerol stocks and grown on Trypticase Soy agar with 5% sheep blood plates (TSA + 5% SB, Becton Dickinson, USA) at 37 °C, in a microaerophilic environment (GenBox system, BioMérieux, France) over 48 h. After, some colonies were streaked in fresh TSA + 5% SB and incubated another 48 h in the settings above mentioned. Then, a pre-inoculum was prepared in Mueller–Hinton Broth supplemented with 10% (*v*/*v*) heat inactivated fetal bovine serum (MHB + 10% FBS) as reported in [40] and incubated overnight (16–18 h) under microaerophilic conditions, 37 °C and 150 rpm.

### 3.7. Preparation of Nanoparticles Suspensions for Biological Testing

Before starting the antimicrobial tests, all samples of materials silver or bismuth nanoparticles (NPs) deposited on the surface of silica spheres (SiO_2_) were dispersed in deionized water at a concentration of 0.5 g of material per 10 mL of water. The suspensions were prepared immediately before testing by sonication for 3 min with external cooling (25 °C). These suspensions were used in all subsequent biological tests.

### 3.8. Minimum Inhibitory Concentration (MIC) Determination

MIC values were determined using the standard broth microdilution method in cation-adjusted Mueller–Hinton broth (CAMHB, Oxoid), in accordance with Clinical and Laboratory Standards Institute (CLSI) guidelines [41]. A fresh overnight culture was centrifuged, and the bacterial pellet was resuspended in sterile saline to achieve a final inoculum density of approximately 3 × 10^5^ colony-forming units (CFU) mL^−1^.

Serial two-fold dilutions of the tested materials were prepared in sterile 96-well microtiter plates (NEST). Each well received 100 µL of the bacterial suspension to reach a final inoculum of 1.5 × 10^5^ CFU mL^−1^ per well. The plates were incubated at 37 °C for 20 h under aerobic conditions with gentle shaking at 100 rpm. After incubation, MIC values were determined using the 3-(4,5-dimethylthiazol-2-yl)-2,5-diphenyl tetrazolium bromide (MTT) colorimetric assay. MTT (Sigma-Aldrich) was added to each well at a final concentration of 0.2 mg mL^−1^ as reported in [42]. Plates were further incubated to allow color development, and the MIC was defined as the lowest concentration that prevented visible color change. All MIC determinations were performed in duplicate across three independent experiments.

For *H. pylori* J99 MIC determination, the overnight culture was adjusted to 2 × 10^7^ CFU mL^−1^ in accordance with the Clinical & Laboratory Standards Institute (CLSI) guidelines [43]. Two ten-fold dilutions of the tested materials (C1 and C2) were prepared in sterile 96-well microtiter plates (NEST). Each well received 100 µL of the bacterial suspension to reach a final inoculum of 1 × 10^7^ CFU mL^−1^ per well. Plates were incubated at 37 °C for 24 h under microaerophilic conditions. The MIC was determined in MHB + 10% FBS using the above-mentioned MTT protocol. MIC determination was performed in triplicate in two independent experiments.

### 3.9. Minimum Bactericidal Concentration (MBC) Determination

MBC was defined as the lowest concentration at which no bacterial growth was observed, and the MBC values were determined following MIC evaluation. From each well showing no visible growth (qualitatively accessed with the MTT assay), 10 µL of the bacterial suspension was plated onto Mueller–Hinton agar (Oxoid Ltd., Basingstoke, UK) and incubated aerobically at 37 °C for 20 h. MBC testing was performed in three independent experiments.

For *H. pylori* J99, following MIC determination, 10 µL samples were collected, serially diluted by 10-fold in phosphate-buffer saline (PBS, 0.1 M), and each dilution was plated in TSA + 5% SB. The plates were incubated at 37 °C for 5 days under microaerophilic conditions and two independent experiments were performed using triplicates.

### 3.10. Cytotoxicity Against Gastric Cell Lines

The most promising nanoparticles against *H. pylori* J99 were further characterized for their cytotoxicity against the human gastric adenocarcinoma cell lines AGS (ATCC^®^ CRL-1739™) and MKN28 (JCRB0253™) by a direct contact assay in accordance with ISO 10993-5 and the sample preparation and reference materials guidance of ISO 10993-12 [44,45]. Cells were cultured in Roswell Park Memorial Institute (RPMI) 1640 medium with Glutamax and 4-(2-hydroxyethyl)-1-piperazineethanesulfonic acid (HEPES) supplemented with 10% fetal bovine serum (FBS) and 1% (*v*/*v*) penicillin-streptomycin (all from Gibco, Thermo Fisher Scientific, Waltham, MA, USA), at 37 °C in a 5% CO_2_ atmosphere. Cell medium was regularly changed until confluence (80–90%) was reached. Then, cells were trypsinized (Trypsin, Sigma-Aldrich, St. Louis, MO, USA), adjusted to a density of 1 × 10^4^ cells mL^−1^ and seeded in a 96-well tissue culture polystyrene plate (TCPS, Sarstedt, Nümbrecht, Germany) for 24 h, at 37 °C and 5% CO_2_. The following day, nanoparticles were centrifuged (3000× *g*, Room Temperature, 10 min) and resuspended in cell culture medium. A 10-fold dilution (C2) was also performed. Then, the culture medium was replaced by the NP solution (C1 and C2) and incubated for another 24 h under the same settings. Cell metabolic activity was evaluated with resazurin colorimetric assay. For that, a resazurin solution (20% *v*/*v*) was added to the wells and incubated for 4 h at 37 °C, 5% CO_2_. After, the supernatant was transferred to a black polypropylene 96-well plate (Greiner, Bio-One GmbH, Frickenhausen, Germany) and fluorescence was measured at a wavelength of 530/590 nm in a microplate fluorometer (Synergy Mx, BioTek Instruments, Inc., Winooski, VT, USA). Cells with only culture medium and cells exposed to 1 mM hydrogen peroxide (H_2_O_2_, 30 vol., Merck KGaA, Darmstadt, Germany) were used as negative and positive control, respectively. Cell viability was expressed in percentage of metabolic activity of nanoparticle-exposed cells in relation to cells of the negative control (cells in culture media). One experiment per cell line with duplicates was performed.

### 3.11. Statistical Analysis

Statistical analysis was performed using GraphPad Prism 10.0 (GraphPad Software, San Diego, CA, USA), using One-way ANOVA followed by Kruskal–Wallis comparison test. Data were expressed as mean ± standard deviation (SD). Statistically significant differences were considered for *p* < 0.05.

## 4. Conclusions

The results of the conducted research indicate the significant application potential of using the antibacterial properties of silica spheres functionalized with silver and bismuth against antibiotic-resistant bacterial strains. Structural, morphological, and biological studies have confirmed that the appropriate surface functionalization of silica spheres with APTES, along with a properly planned synthesis sequence and process conditions, enables effective metal deposition on the silica surface and stabilization of nanostructures, resulting in strong antimicrobial activity.

The use of silica as a carrier effectively limited the aggregation of silver and bismuth nanoparticles deposited on its surface and enabled their stable and controlled deposition. Surface functionalization of silica spheres with amino groups (APTES) promoted a more uniform distribution of metal nanoparticles and increased colloidal stability. However, this did not enhance antibacterial activity because the antimicrobial performance was governed primarily by the release and availability of bioactive silver species rather than by nanoparticle dispersion or size. The presence of amino groups on the silica surface may have partially limited the accessibility of these species, thereby counteracting the potential benefits of increased surface area and more uniform nanoparticle distribution.

For the bismuth-based nanocomposites, no clear advantage of APTES functionalization on antimicrobial effectiveness was observed, and the sample without surface modification, SiO_2_-BiNPs1, in which bismuth was present mainly in an amorphous or poorly ordered form, showed comparable or in some cases even higher activity against most bacterial strains. In the SiO_2_-BiNPs2 sample, which contained the identified bismuth oxide-nitrate hydrate phase (Bi_6_O_5_(OH)_3_(NO_3_)_5_·3H_2_O), the presence of this phase may have contributed to slow and gradual release of Bi^3+^ ions, potentially modulating antibacterial performance. Tests against *Helicobacter pylori* J99 showed complete inhibition of bacterial growth in the presence of both SiO_2_-BiNP variants, confirming the potential of bismuth-functionalized nanocomposites in combating gastrointestinal pathogens.

The results of SEM, TEM, and XRD analyses confirmed the differences in the nature of crystalline and amorphous metal phases deposited on the SiO_2_ surface, and that the presence of PVP and APTES may play a significant role in shaping the morphology and kinetics of ion release from the surface of silica spheres.

Zeta potential measurements confirmed negative values for all synthesized materials, which suggests that electrostatic attraction is unlikely to be the dominant driver of activity. Ion release combined with multi-target surface interactions likely contributes substantially to the observed antibacterial effects.

## Figures and Tables

**Table 1:** Composition and surface modification of silica spheres functionalized with silver or bismuth nanoparticles: planned and obtained metal content (wt.% relative to SiO_2_).

Sample	Metal	Modification Description	Planned [wt.%]	Obtained * [wt.%]
SiO_2_-AgNPs1	Ag	Silica spheres with silver nanoparticles, no APTES	3.0	2.04
SiO_2_-AgNPs2	Ag	Silica spheres with silver nanoparticles, with APTES	3.0	2.57
SiO_2_-BiNPs1	Bi	Silica spheres with bismuth nanoparticles, no APTES	3.0	2.38
SiO_2_-BiNPs2	Bi	Silica spheres with bismuth nanoparticles, with APTES	5.0	4.52

* Obtained metal contents determined by ICP-OES are discussed in Results (Phase composition, XRD).

**Table 2:** Minimum inhibitory concentration (MIC) and minimum bactericidal concentration (MBC) of silica spheres functionalized with silver or bismuth nanoparticles against selected Gram-positive and Gram-negative bacterial strains. All MIC and MBC values are expressed in µg mL^−1^.

Sample	Tested Strains													
	*S. aureus* ATCC 25923		*S. aureus* ATCC 43300 MRSA		*S. epidermidis* ATCC 28212		*E. faecium* VRE ATCC 700221		*E. faecalis* ATCC 29212		*E. coli* ATCC 25922		*P. aeruginosa* ATCC 27853	
	MIC	MBC	MIC	MBC	MIC	MBC	MIC	MBC	MIC	MBC	MIC	MBC	MIC	MBC
SiO_2_-AgNPs1 Ag 1020 µg mL^−1^	c2	c1	c2	c1	c3	c1	c1–c2	>c1	c1	>c1	c1	>c1	>c1	not tested
	255.0	510.0	255.0	510.0	127.5	510.0	382.5	510.0	510.0	510.0	510.0	510.0	>510.0	not tested
SiO_2_-AgNPs2 Ag 1285 µg mL^−1^	c2	c1	c2	c1	c3	c1	c2	>c1	c2	>c1	c1	>c1	>c1	not tested
	321.2	642.5	321.2	642.5	160.6	642.5	321.2	642.5	321.2	642.5	642.5	642.5	>642.5	not tested
SiO_2_-BiNPs1 Bi 1190 µg mL^−1^	c2	>c1	c2	c1	c4	c2–c3	c2	>c1	c2	c1	c1	>c1	>c1	not tested
	297.5	>595	297.5	>595	74.375	297.5–148.75	297.5	>595	297.5	595	595	>595	>595	not tested
SiO_2_-BiNPs2 Bi 2260 µg mL^−1^	c3	c2	c3	c1	c5	c3	c2	>c1	c2–c3	c1	c1	>c1	>c1	not tested
	282.5	565	282.5	1130	70.625	282.5	565	1130	423.75	1130	1130	1130	>1130	not tested
Control	No inhibition	No inhibition	No inhibition	No inhibition	No inhibition	No inhibition	No inhibition	No inhibition	No inhibition	No inhibition	No inhibition	No inhibition	No inhibition	not tested

## Data Availability

The data presented in this study are available within the article.

## Abbreviations

The following abbreviations are used in this manuscript:

AgNPs	Silver nanoparticles
APTES	(3-Aminopropyl)triethoxysilane
BiNPs	Bismuth nanoparticles
CFU	Colony-forming unit
CLSI	Clinical and Laboratory Standards Institute
EDS	Energy-dispersive X-ray spectroscopy
EtOH	Ethanol
FCC	Face-centered cubic
FBS	Fetal bovine serum
H_2_O_2_	Hydrogen peroxide
ICP-OES	Inductively coupled plasma–optical emission spectrometry
ISO	International Organization for Standardization
MIC	Minimum inhibitory concentration
MBC	Minimum bactericidal concentration
MDR	Multidrug resistant
MRSA	Methicillin-resistant *Staphylococcus aureus*
NH_4_OH	Ammonium hydroxide
NPs	Nanoparticles
PBS	Phosphate-buffered saline
PES	Polyethersulfone
PVP	Polyvinylpyrrolidone
ROS	Reactive oxygen species
RPMI	Roswell Park Memorial Institute medium
SEM	Scanning electron microscopy
SiO_2_	Silica
TEOS	Tetraethoxysilane
TEM	Transmission electron microscopy
VRE	Vancomycin-resistant Enterococcus
WHO	World Health Organization
XRD	X-ray diffraction
